# Supplementary material for: Cell division drives DNA methylation loss in late-replicating domains in primary human cells
Source: Nat Commun. 2022 Nov 8;13:6659. doi: 10.1038/s41467-022-34268-8 (PMC9643452; doi:10.1038/s41467-022-34268-8)
Supplement: Supplementary file 1 — Supplementary Information [file 41467_2022_34268_MOESM1_ESM.pdf]

Supplementary Material for:

Cell division drives DNA methylation loss in late-replicating domains in primary human cells

Jamie L. Endicott, Paula A. Nolte, Hui Shen*, Peter W. Laird*Department of Epigenetics, Van Andel Institute, Grand Rapids, MI.

Nature Communications

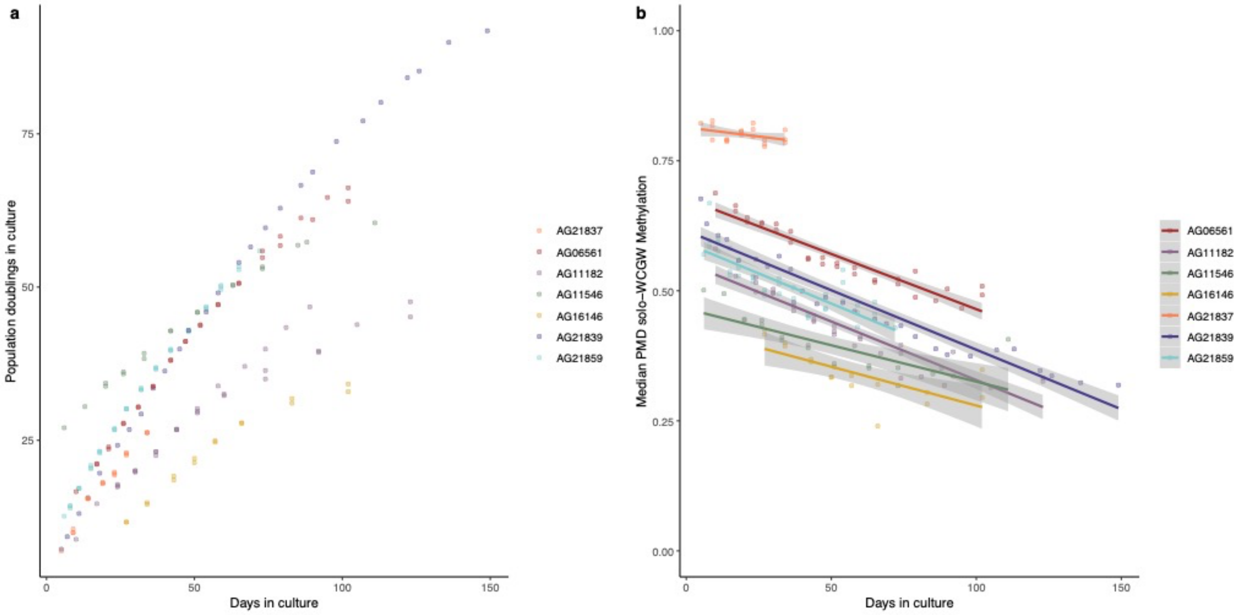

Supplementary Figure 1

Correlation of median PMD solo-WCGW methylation with time. **a**, Growth curves of cell lines used in this study. **b**, Plot of median fractional methylation at 26,732 PMD solo-WCGWs present on InfiniumEPIC Methylation array vs. days in culture during this study. Regression lines are linear with shaded regions representing 95% confidence interval.

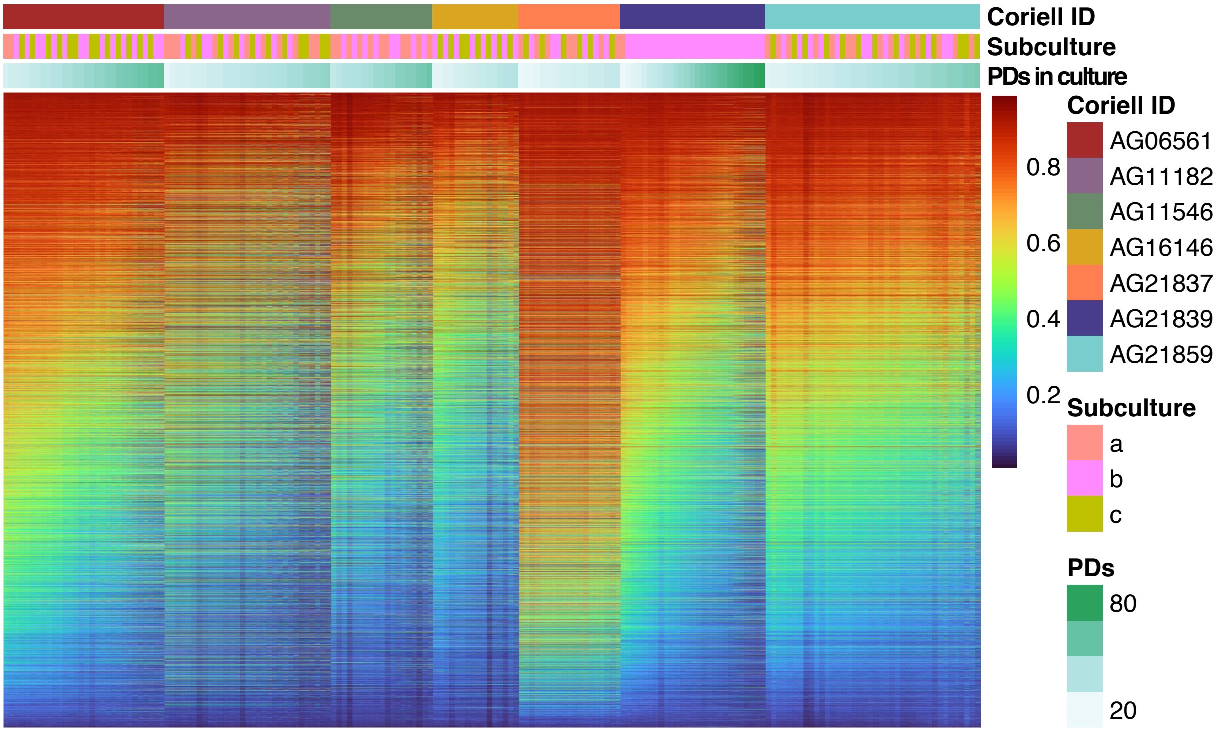

Supplementary Figure 2
Heatmap of PMD solo-WCGW methylation for primary cell lines included in this study. Rows (CpGs) are ordered by the mean of each cell line's median methylation value across timepoints profiled. Columns (samples) are ordered by advancing population doublings (PDs).

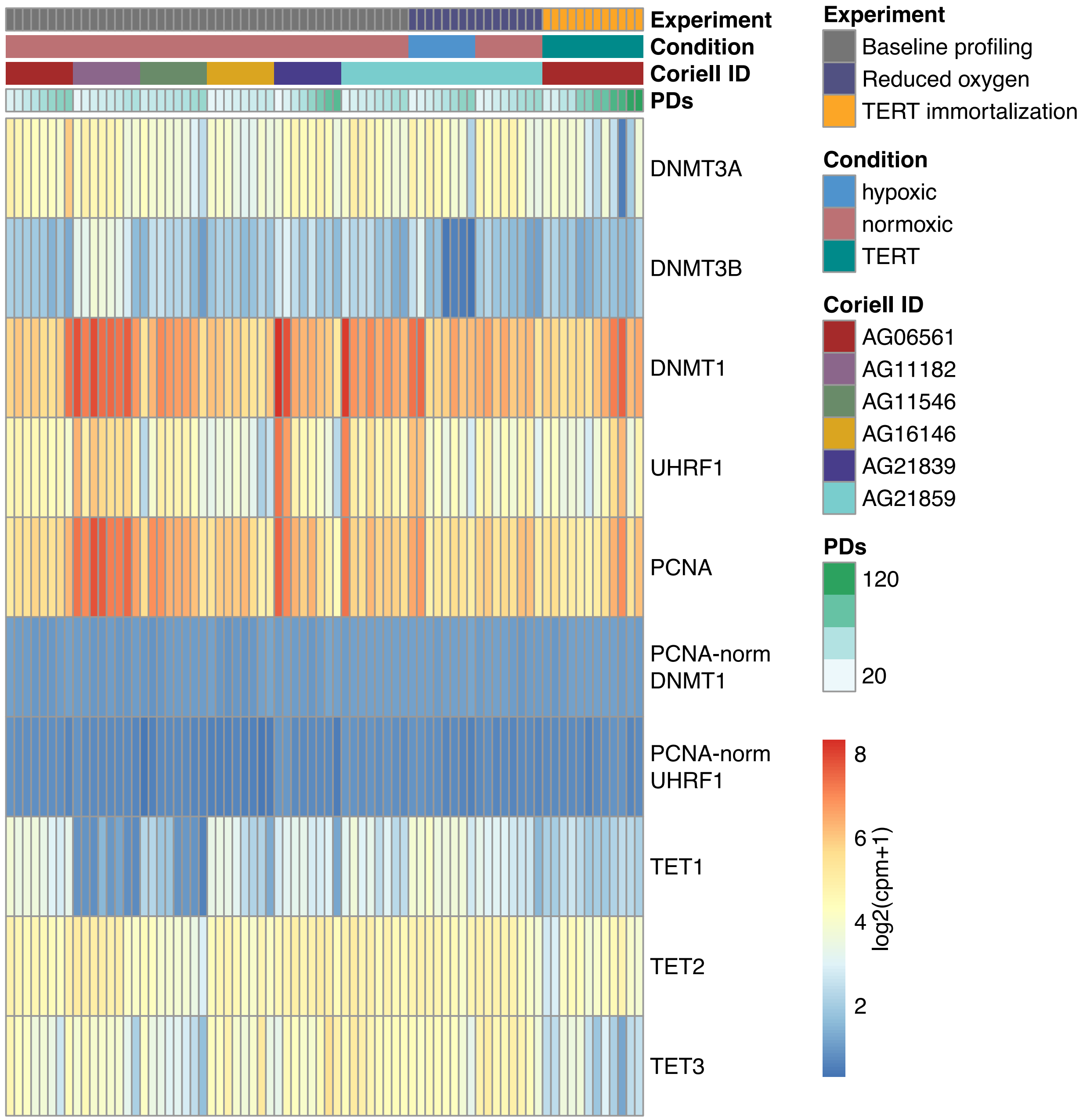

Supplementary Figure 3
Gene expression heatmap of key enzymes involved in DNA methylation patterning. Columns (samples) are ordered first by experiment, then cell line, then by advancing population doublings (PDs).

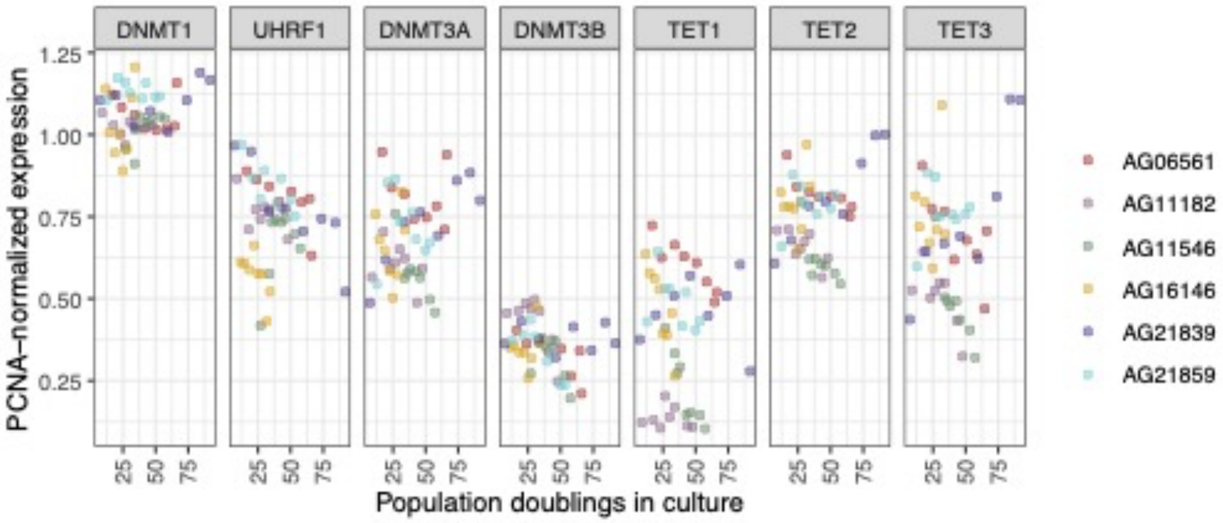

Supplementary Figure 4
PCNA-normalized gene expression of key enzymes involved in DNA methylation patterning vs population doublings in culture.

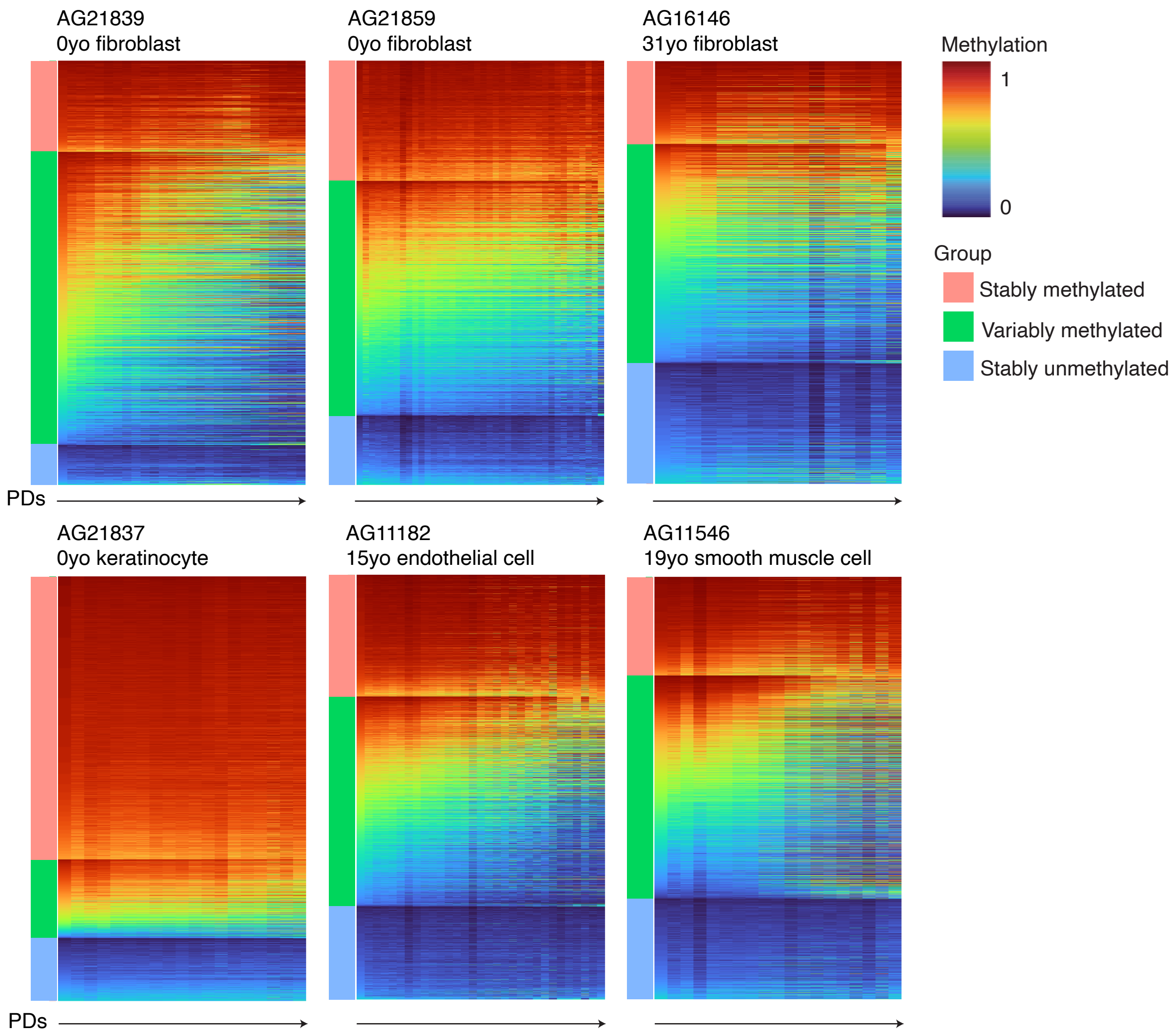

Supplementary Figure 5
Methylation heatmaps of PMD solo-WCGWs. CpGs were separated into major categories: (from top) stably methylated, variably methylated, and stably unmethylated. Samples (columns) are ordered by advancing population doublings (PDs).

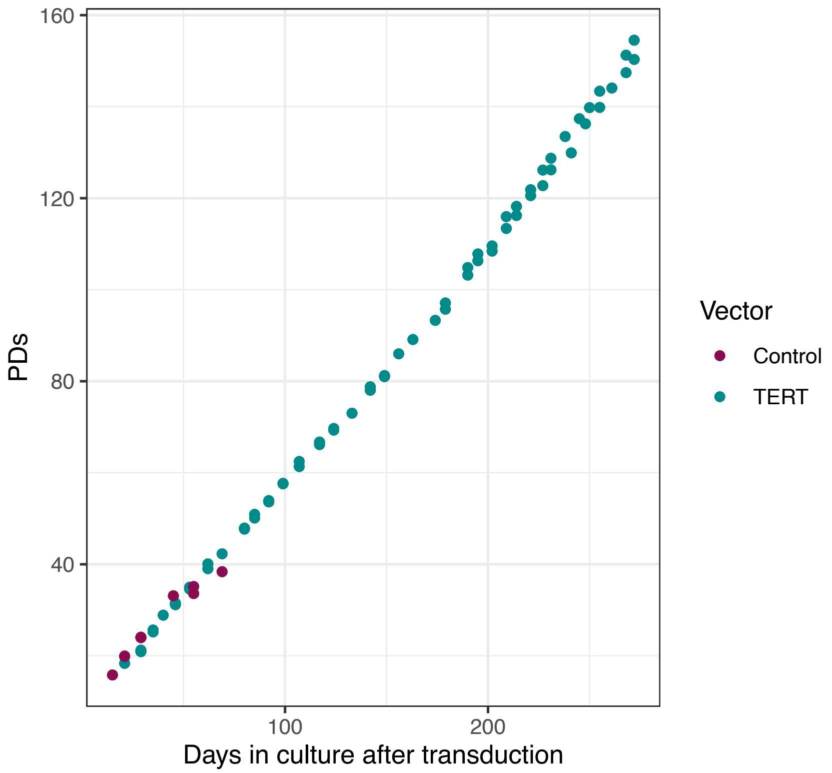

Supplementary Figure 6

Growth curve of *TERT*-immortalized primary skin fibroblasts (AG06561). Control vector fibroblasts (dark pink) senesced at approximately 40 population doublings (PDs); at the time of analysis *TERT*-immortalized cells (blue) remained highly proliferative.

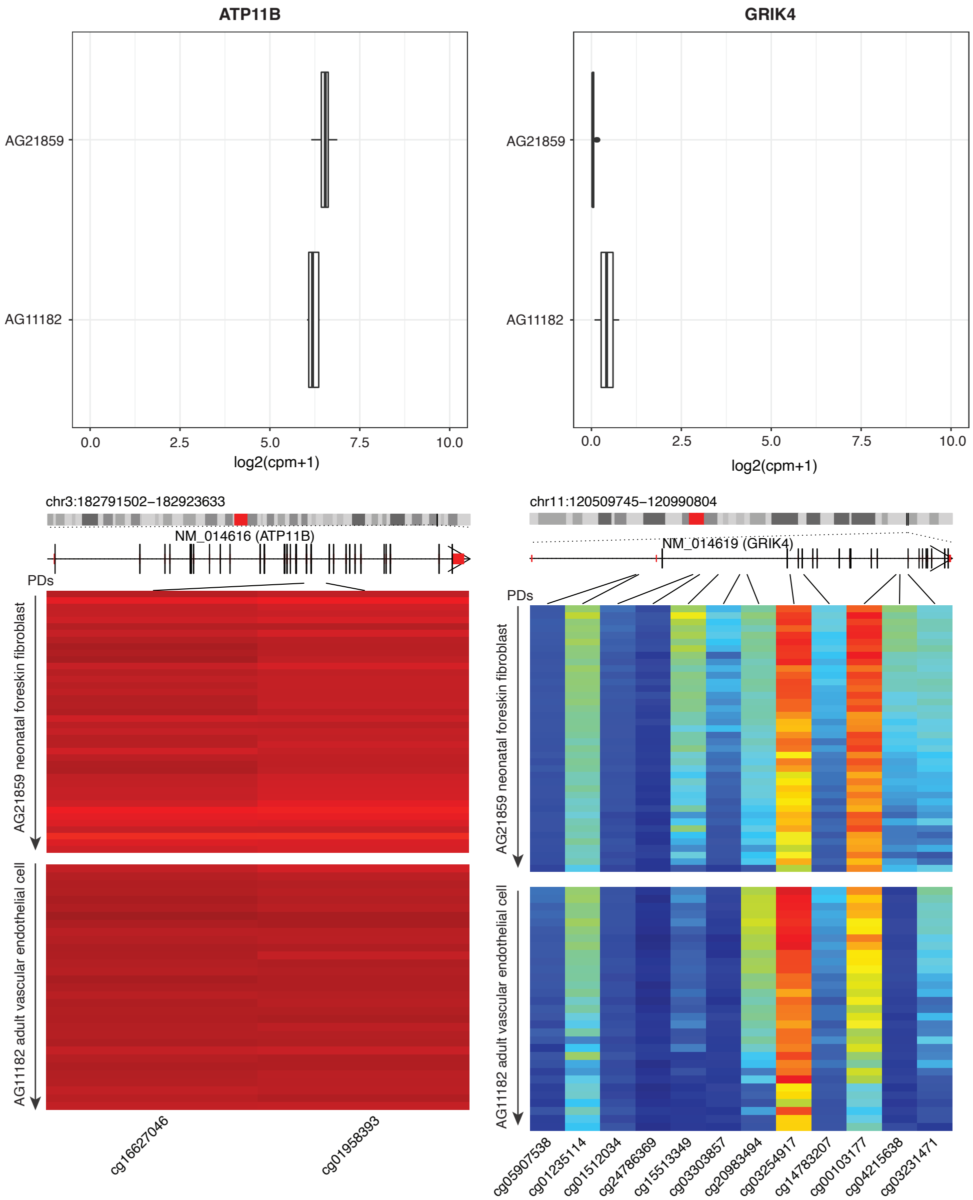

Supplementary Figure 7
Gene expression protects against replication-associated methylation loss. Upper panels: boxplots of normalized gene expression for similarly expressed genes: *ATP11B* (left) and *GRIK4* (right). Boxplots depict data quartiles; center bar depicts median value. Lower panels: methylation at gene-associated PMD solo-WCGWs. Samples (rows) are arranged from early PD to late PD.

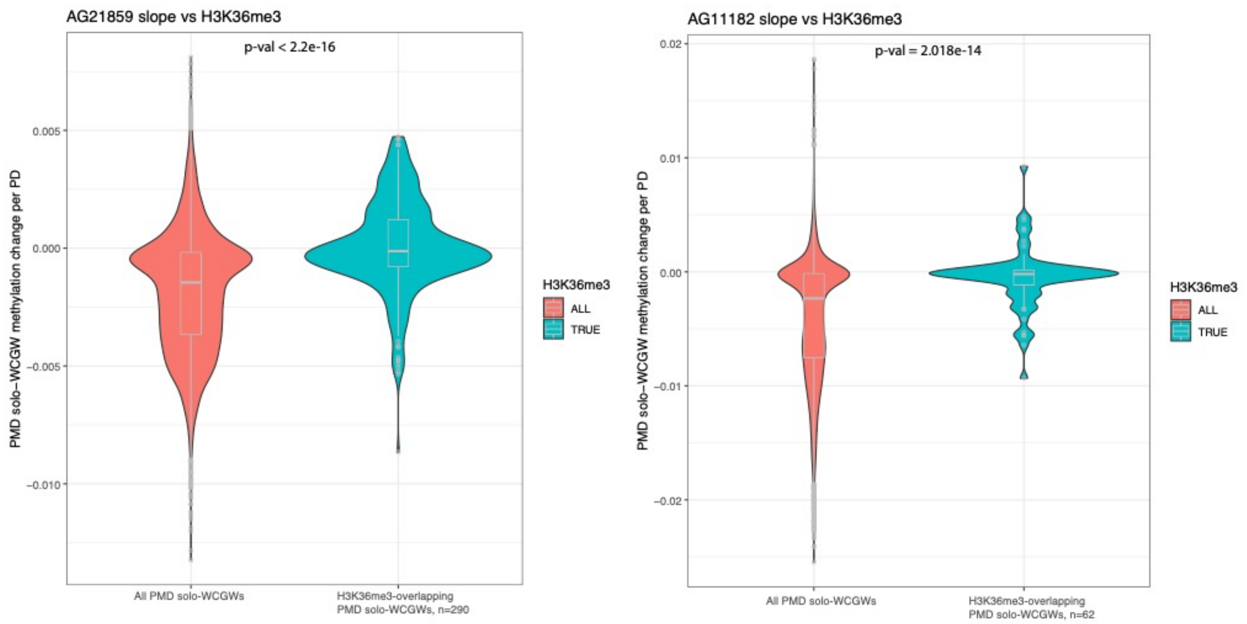

Supplementary Figure 8
H3K36me3 protects against replication-associated methylation loss.
CpG-wise methylation change per PD for PMD solo-WCGWs overlapping H3K36me3 for primary fibroblasts (AG21859, left) and endothelial cell (AG11182, right). Boxplots depict data quartiles; center bar depicts median value. Statistical comparison by two-sided Kruskal-Wallis test.

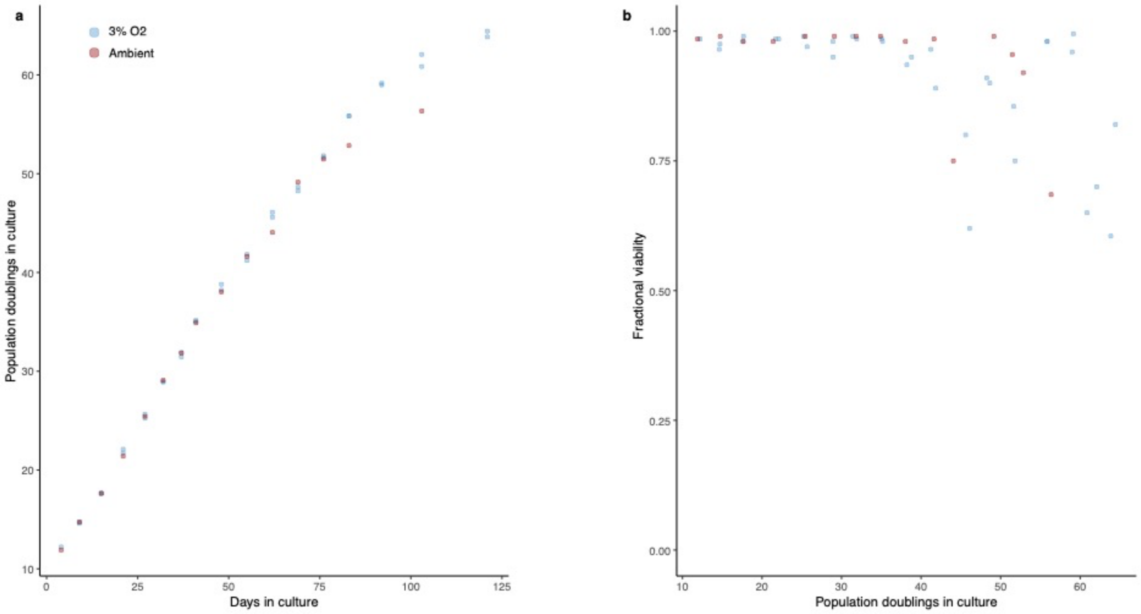

Supplementary Figure 9
a, Growth curves of primary fibroblast (AG21859) grown under ambient oxygen conditions (pink) and low 3% oxygen (blue). **b**, Cellular viability of primary fibroblasts under both oxygen culture conditions.

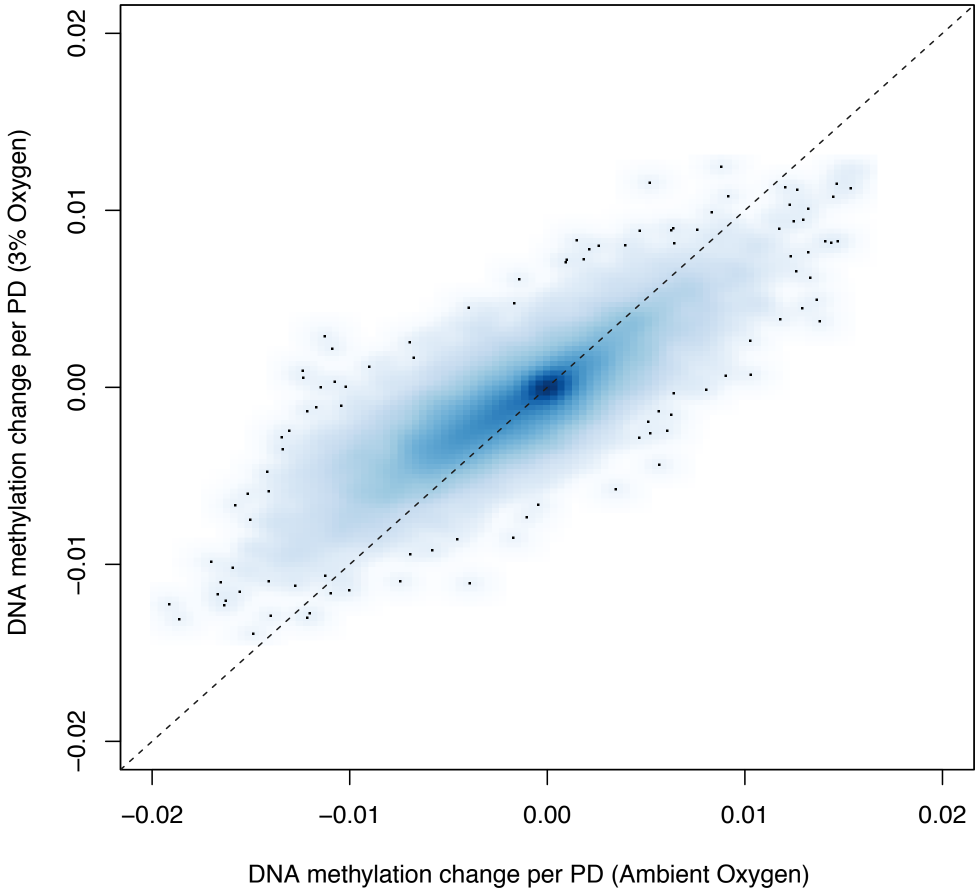

Supplementary Figure 10
Comparison of PMD solo-WCGW methylation change per population doubling (PD) under 3% oxygen culture vs ambient oxygen culture. Dotted line represents an identity line of y=x.

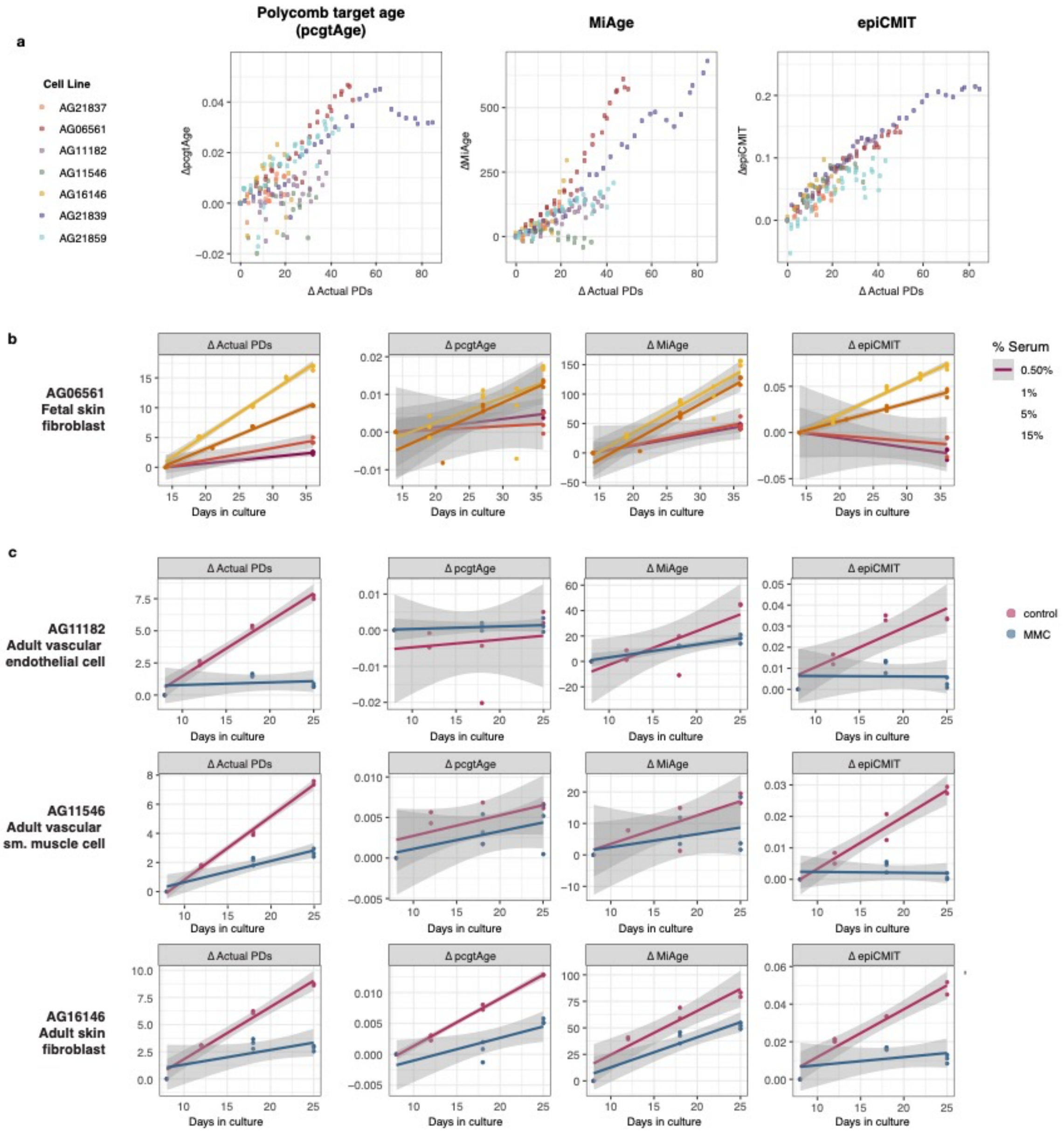

Supplementary Figure 11 | Performance of other mitotic clocks on primary DNA methylation data

Clock performance on (**a**) primary human cells cultured under standard conditions through replicative senescence; (**b**) primary human fibroblasts grown under different % v/v media serum to encourage differential proliferation rates; (**c**) primary human cells treated with DNA replication inhibitor Mitomycin C (MMC) or vehicle control. Solid lines depict linear regression with gray shading depicting 95% confidence interval.

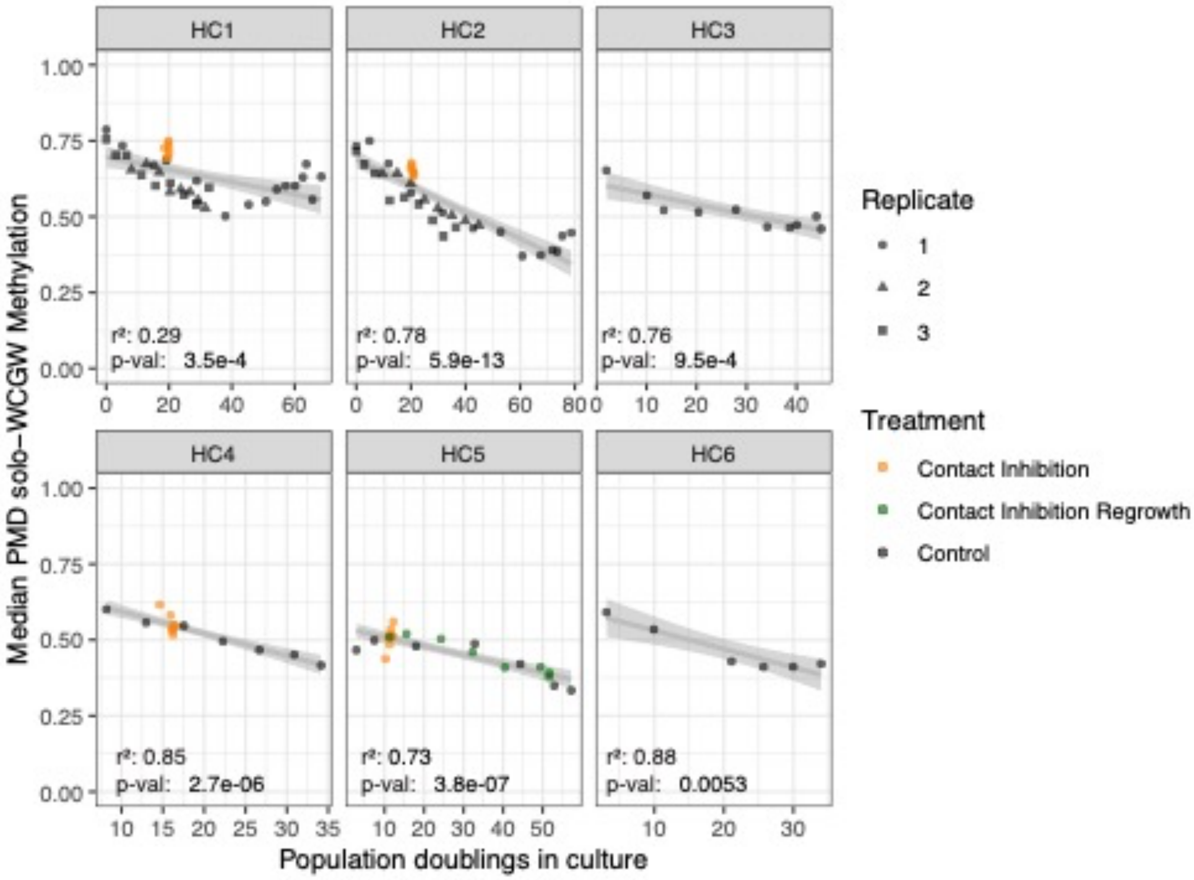

Supplementary Figure 12 | Loss of PMD solo-WCGW methylation as a consequence of cellular replicative history, external DNA methylation dataset GSE179847. Solid lines depict linear regression with gray shading depicting 95% confidence interval; statistical analyses are two-sided.

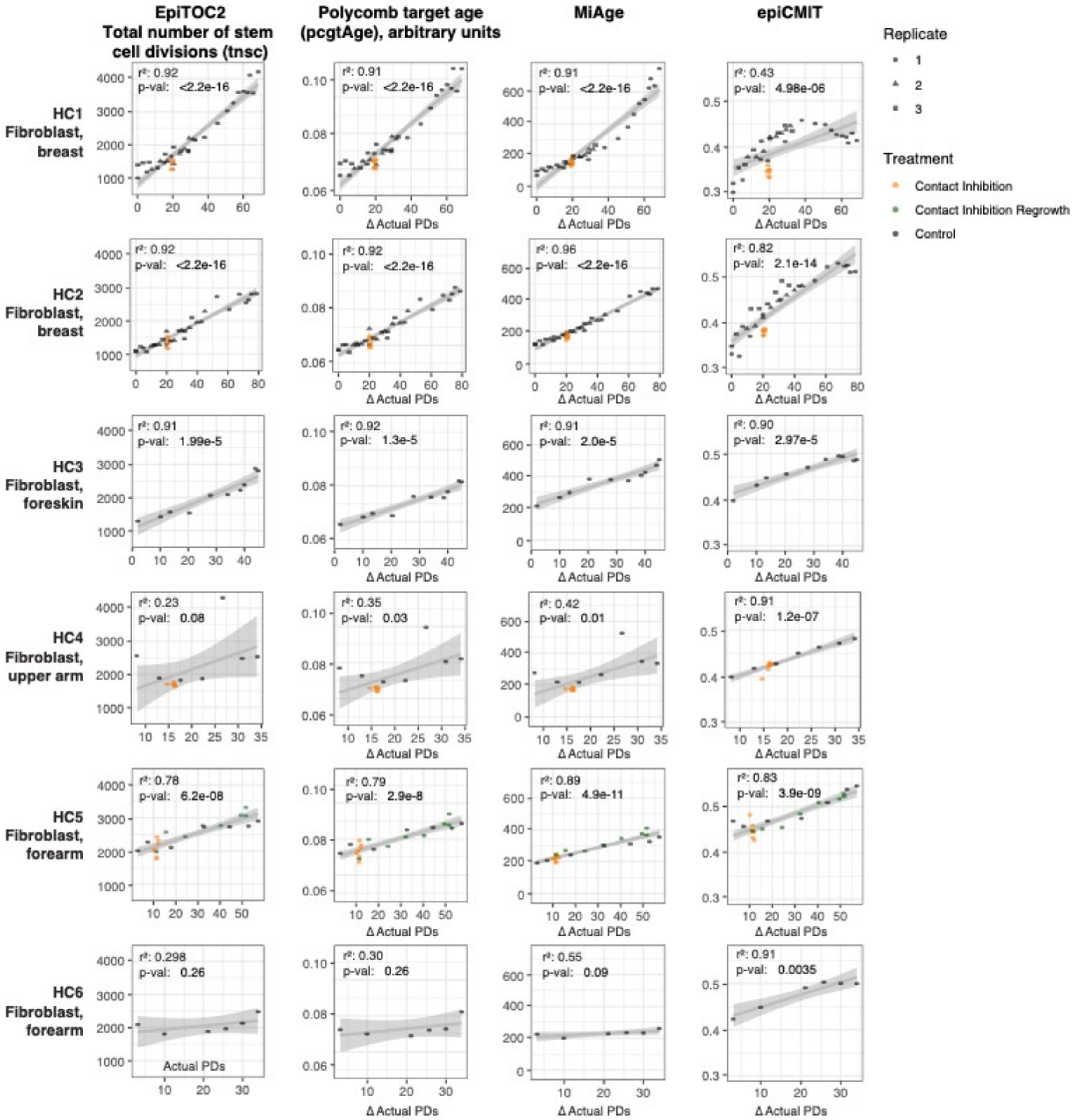

Supplementary Figure 13 | Performance of other mitotic clocks on external DNA methylation dataset GSE179847. Solid lines depict linear regression with gray shading depicting 95% confidence interval; statistical analyses are two-sided.

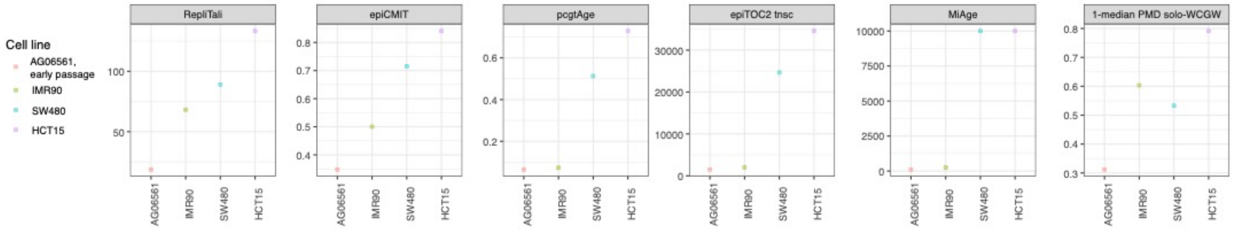

Supplementary Figure 14 | Mitotic clock estimates of replicative age on well-classified human cell lines. AG06561: Primary human fibroblast, early passage, for comparison. **IMR90**: Fetal lung fibroblast-derived cell line. **SW480**: Colon adenocarcinoma-derived cell line. **HCT15**: Colon adenocarcinoma-derived cell line.

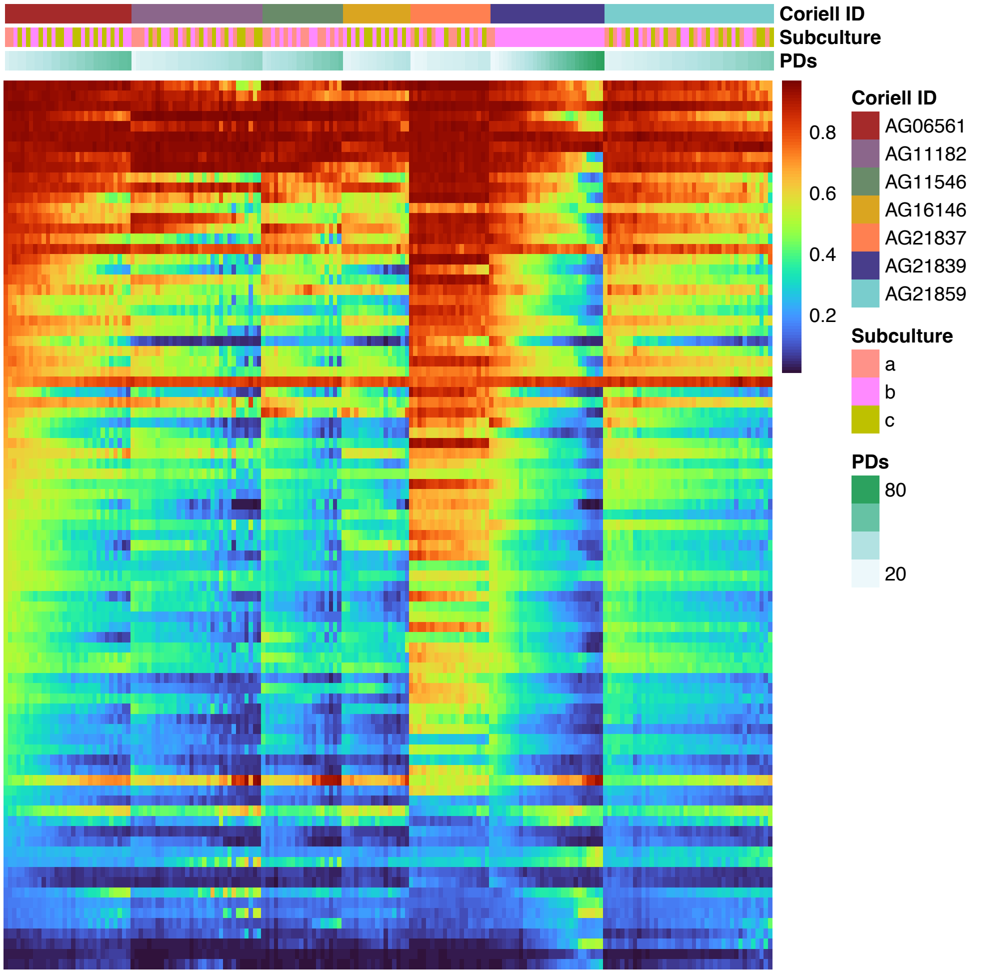

Supplementary Figure 15 | RepliTali CpG methylation for serially passaged cells.

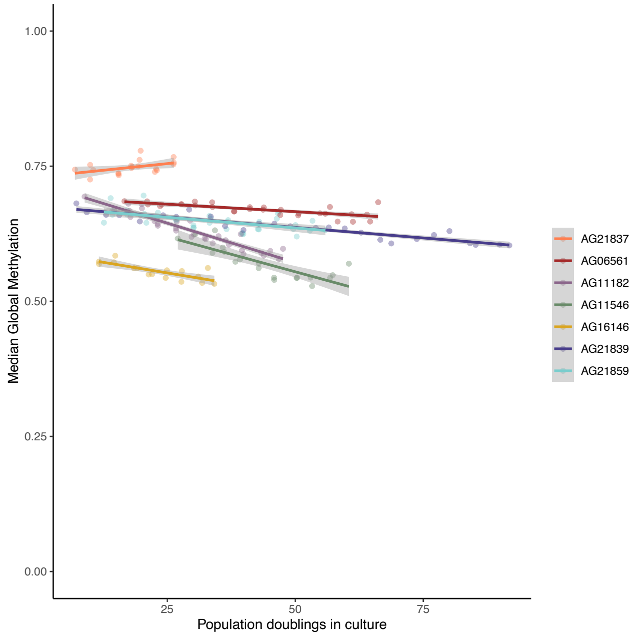

Supplementary Figure 16: Global methylation in serially passaged primary cells grown under standard culture conditions. Solid lines depict linear regression with gray shading depicting 95% confidence interval.

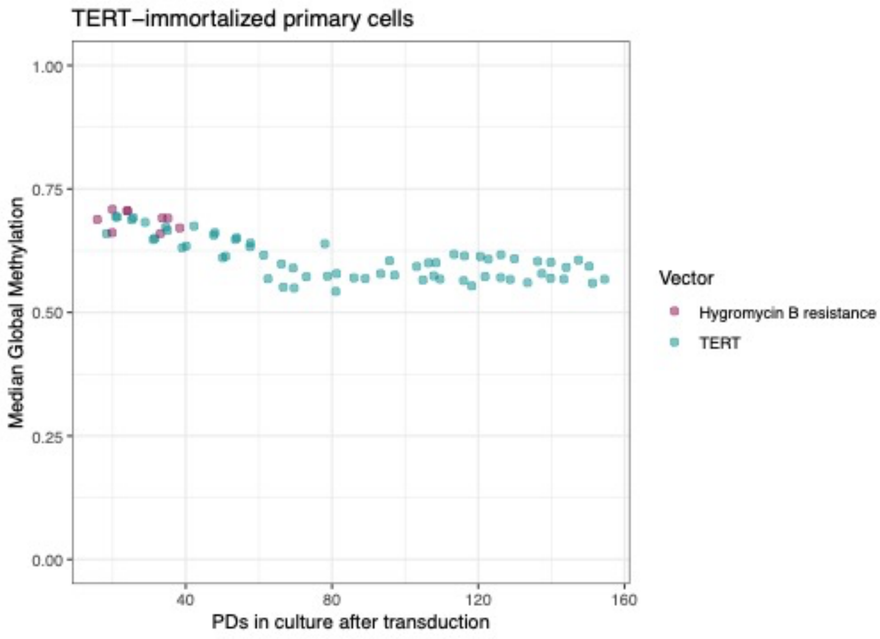

Supplementary Figure 17: Global DNA methylation in immortalized primary fibroblast AG06561.

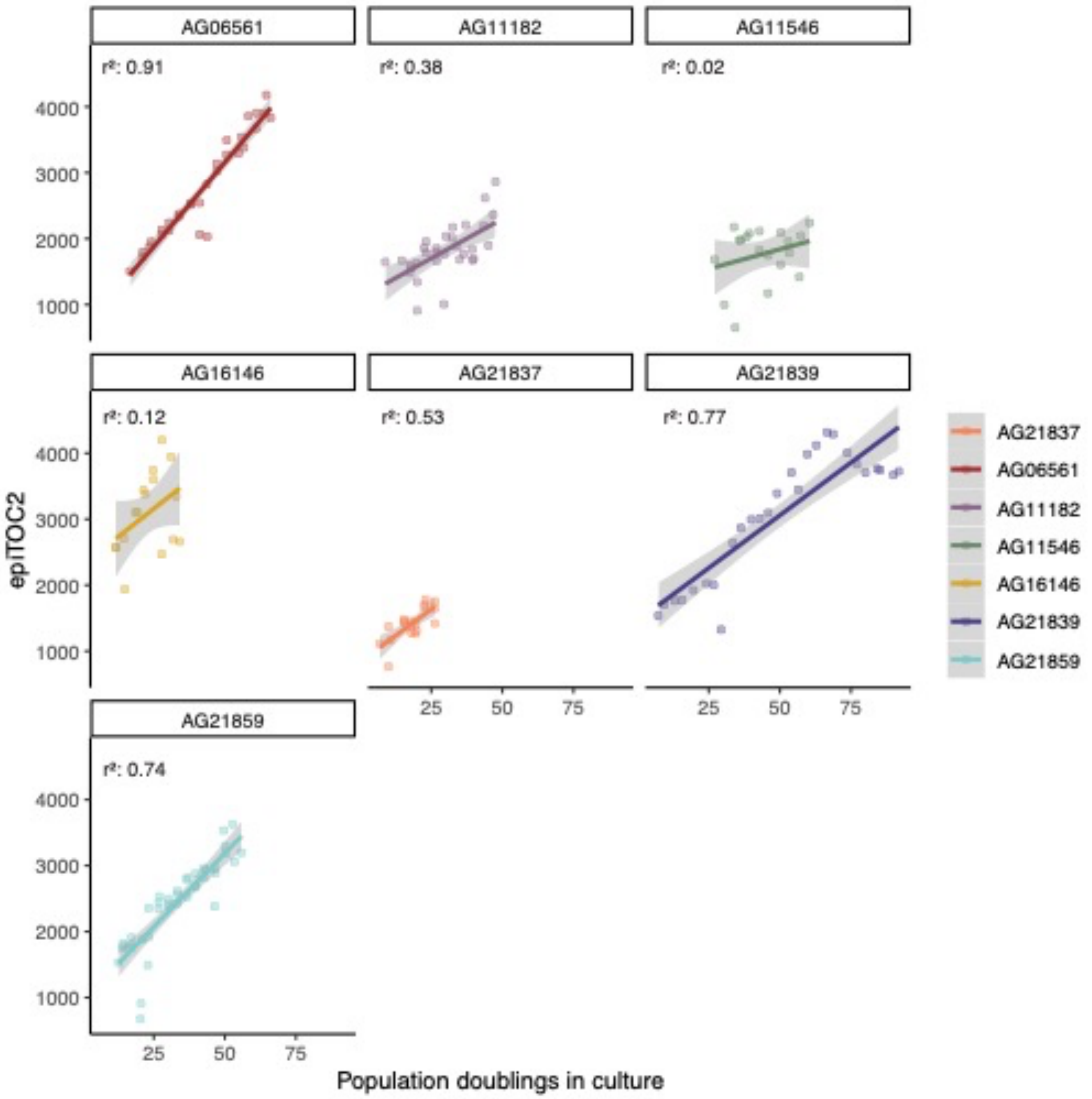

Supplementary Figure 18: epiTOC2 estimate by primary cell. Solid lines depict linear regression with gray shading depicting 95% confidence interval; statistical analyses are two-sided.

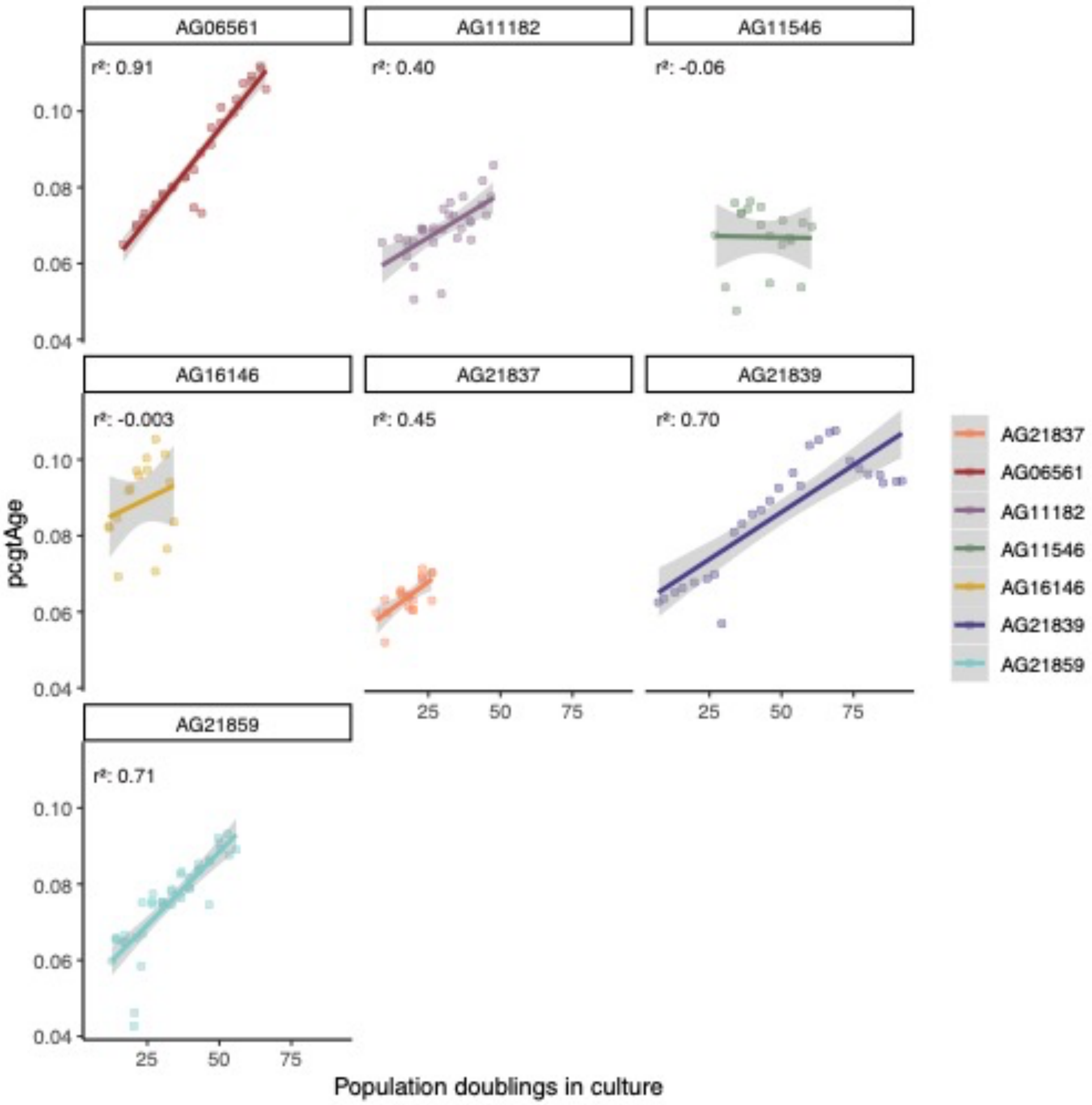

Supplementary Figure 19: pcgtAge estimate by primary cell. Solid lines depict linear regression with gray shading depicting 95% confidence interval; statistical analyses are two-sided.

Contents of additional materials:

Supplementary Data 1: Summary of primary cell cultures used in this study.

Supplementary Data 2: Groupwise Locus Overlap Enrichment Analysis results for TERT-immortalized fibroblasts

Supplementary Data 3: Differential gene expression analysis for low oxygen vs ambient oxygen culture conditions

Supplementary Data 4: RepliTali coefficients

Supplementary Data 5: GSE179847 characteristics
