# Supplementary material for: Cell division drives DNA methylation loss in late-replicating domains in primary human cells
Source: Nat Commun. 2022 Nov 8;13:6659. doi: 10.1038/s41467-022-34268-8 (PMC9643452; doi:10.1038/s41467-022-34268-8)
Supplement: Supplementary file 3 — Description of additional Supplementary File [file 41467_2022_34268_MOESM3_ESM.pdf]

Descriptions of additional Supplementary Files

Supplementary Data 1: Summary of primary cell cultures used in this study.
Supplementary Data 2: Groupwise Locus Overlap Enrichment Analysis results for TERT-immortalized fibroblasts
Supplementary Data 3: Differential gene expression analysis for low oxygen vs ambient oxygen culture conditions
Supplementary Data 4: RepliTali coefficients
Supplementary Data 5: GSE179847 characteristics
