# Supplementary material for: Cell division drives DNA methylation loss in late-replicating domains in primary human cells
Source: Nat Commun. 2022 Nov 8;13:6659. doi: 10.1038/s41467-022-34268-8 (PMC9643452; doi:10.1038/s41467-022-34268-8)
Supplement: Supplementary file 9 — Reporting Summary [file 41467_2022_34268_MOESM9_ESM.pdf]

nature portfolio

Corresponding author(s): Peter W Laird
Last updated by author(s): Sept 24th, 2022

Reporting Summary

Nature Portfolio wishes to improve the reproducibility of the work that we publish. This form provides structure for consistency and transparency in reporting. For further information on Nature Portfolio policies, see our Editorial Policies and the Editorial Policy Checklist.

Statistics

For all statistical analyses, confirm that the following items are present in the figure legend, table legend, main text, or Methods section.

n/a	Confirmed	
☐	☒	The exact sample size (*n*) for each experimental group/condition, given as a discrete number and unit of measurement
☐	☒	A statement on whether measurements were taken from distinct samples or whether the same sample was measured repeatedly
☐	☒	The statistical test(s) used AND whether they are one- or two-sided<br>*Only common tests should be described solely by name; describe more complex techniques in the Methods section.*
☐	☒	A description of all covariates tested
☐	☒	A description of any assumptions or corrections, such as tests of normality and adjustment for multiple comparisons
☐	☒	A full description of the statistical parameters including central tendency (e.g. means) or other basic estimates (e.g. regression coefficient) AND variation (e.g. standard deviation) or associated estimates of uncertainty (e.g. confidence intervals)
☐	☒	For null hypothesis testing, the test statistic (e.g. *F*, *t*, *r*) with confidence intervals, effect sizes, degrees of freedom and *P* value noted<br>*Give P values as exact values whenever suitable.*
☒	☐	For Bayesian analysis, information on the choice of priors and Markov chain Monte Carlo settings
☐	☒	For hierarchical and complex designs, identification of the appropriate level for tests and full reporting of outcomes
☒	☐	Estimates of effect sizes (e.g. Cohen's *d*, Pearson's *r*), indicating how they were calculated

Our web collection on statistics for biologists contains articles on many of the points above.

Software and code

Policy information about availability of computer code

Data collection	DNA Methylation arrays were imaged using BeadArray Reader using standard recommended Illumina scanner setting.<br>RNA-seq: Pooled libraries were sequenced using 2x50 bp sequencing on the Illumina NovaSeq 6000 sequencer at the Van Andel Institute. Data were demultplexed using Bcl2fastq v1.9.0.
Data analysis	Custom code used in this study is deposited here: https://github.com/jamieendicott/Nature_Comm_2022

For manuscripts utilizing custom algorithms or software that are central to the research but not yet described in published literature, software must be made available to editors and reviewers. We strongly encourage code deposition in a community repository (e.g. GitHub). See the Nature Portfolio guidelines for submitting code & software for further information.

Data

Policy information about availability of data

All manuscripts must include a data availability statement. This statement should provide the following information, where applicable:

- Accession codes, unique identifiers, or web links for publicly available datasets
- A description of any restrictions on data availability
- For clinical datasets or third party data, please ensure that the statement adheres to our policy

The DNA methylation array and RNA sequencing data generated in this study have been deposited in the Gene Expression Omnibus under SuperSeries accession GSE197545 [https://0-www-ncbi-nlm-nih-gov.brum.beds.ac.uk/geo/query/acc.cgi?acc=GSE197545]. This accession includes both raw and processed data. Source

data are provided with this paper in supplementary file Source_Data.xlsx. External public datasets used in this study are listed below:

InfiniumEPIC Methylation probe manifest66 is available here:
https://zwdzwd.github.io/InfiniumAnnotation
Common PMD coordinates, as well as coordinates and characteristics of PMD solo-WCGWs genome-wide and present on the InfiniumEPIC Methylation array are documented here:
https://zwdzwd.github.io/pmd

Replication timing
Replication timing data from BJ foreskin fibroblasts and HUVECs was generated by the University of Washington and maintained by ENCODE. Files are available here:
http://genome.ucsc.edu/cgi-bin/hgFileUi?db=hg19&g=wgEncodeUwRepliSeq
Replication timing weighted average (WA) scores were calculated as previously specified67:
(1) WA=(0.917*G1b)+(0.750*S1)+(0.583*S2)+(0.417*S3)+(0.250*S4)+(0*G2)

H3K36me3
Histone ChIP-seq data from neonatal foreskin fibroblasts was generated by Joseph Costello's lab at UCSF/Roadmap Epigenomics Project. Histone ChIP-seq data from HUVECs was generated by the University of Washington/ENCODE project.
Neonatal foreskin fibroblast: ENCSR889OUV | GSM817238 [https://www.ncbi.nlm.nih.gov/geo/query/acc.cgi?acc=GSM817238].
HUVEC: ENCSR000DVM | GSM945233 [https://www.ncbi.nlm.nih.gov/geo/query/acc.cgi?acc=GSM945233].

DNA Methylation data
Infinium MethylationEPIC array data from serially passaged human fibroblasts was generated by Martin Picard's lab at Colombia University (Cellular Lifespan Study 1.062, GSE179847 [https://www.ncbi.nlm.nih.gov/geo/query/acc.cgi?acc=GSE179847]). Raw idats were reprocessed as above.

Code Availability Statement
Custom code used in this study is deposited here: https://zenodo.org/badge/latestdoi/516036288

Human research participants

Policy information about studies involving human research participants and Sex and Gender in Research.

Reporting on sex and gender	N/A
Population characteristics	N/A
Recruitment	N/A
Ethics oversight	N/A

Note that full information on the approval of the study protocol must also be provided in the manuscript.

Field-specific reporting

Please select the one below that is the best fit for your research. If you are not sure, read the appropriate sections before making your selection.

☒ Life sciences ☐ Behavioural & social sciences ☐ Ecological, evolutionary & environmental sciences

For a reference copy of the document with all sections, see nature.com/documents/nr-reporting-summary-flat.pdf

Life sciences study design

All studies must disclose on these points even when the disclosure is negative.

Sample size	Sample size was not pre-determined by power calculations. Instead, it was biologically informed: for methylation data, each timepoint was profiled when possible, through replicative senescence. We intentionally included multiple cell types (somewhat limited by what is available from cell banks).
Data exclusions	Of 386 DNA methylation samples run, 14 failed quality control during .idat file processing via R package SeSaMe (methods) and were excluded from further analysis.
Replication	Multiple primary cells of different lineages were included when possible to ensure the observed findings were reproducible in a cell-type and technically- independent manner. Experiments were performed in replicate as described in the manuscript. All data are presented. Replicates were successful with minor differences noted in the manuscript. The growth arrest experiment was replicated in primary cells of different types, representing a more complete biological replication.
Randomization	This study consisted mostly of longitudinally collected samples with as many timepoints as logistically possible. Timepoints were randomized

	in the DNA methylation array plate layout to avoid systematic bias in DNA methylation measurements.
Blinding	DNA methylation data was collected using BeadArrays and processed using SeSAMe in an automated fashion, without annotation of the experimental conditions for the samples on the array. Blinding to experimental condition for the experimental steps was not possible due to different cell culture requirements for cell types used, and different conditions such as culture oxygen concentration being directly tested.

Reporting for specific materials, systems and methods

We require information from authors about some types of materials, experimental systems and methods used in many studies. Here, indicate whether each material, system or method listed is relevant to your study. If you are not sure if a list item applies to your research, read the appropriate section before selecting a response.

Materials & experimental systems

n/a	Involved in the study
☒	☐ Antibodies
☐	☒ Eukaryotic cell lines
☒	☐ Palaeontology and archaeology
☒	☐ Animals and other organisms
☒	☐ Clinical data
☒	☐ Dual use research of concern

Methods

n/a	Involved in the study
☒	☐ ChIP-seq
☒	☐ Flow cytometry
☒	☐ MRI-based neuroimaging

Eukaryotic cell lines

Policy information about cell lines and Sex and Gender in Research

Cell line source(s)	All primary cell lines used in this study were obtained from Coriell Institute for Medical Research. Male cell lines used in this study: AG11182, AG11546, AG21839, AG21838, AG21859, AG16146. Female cell line used in this study: AG06561.
Authentication	All primary cell lines used in this study were authenticated by vendor Coriell Institute. Karyotyping was performed on all cells; morphology was noted. For primary cell lines AG11182 and AG11546, additional immunofluorescence assays for cell-type-specific markers was performed.
Mycoplasma contamination	Cell lines used in this study tested negative for mycoplasma via PCR-based assay. Testing was performed by Coriell Institute following recovery from frozen stock.
Commonly misidentified lines (See ICLAC register)	No commonly misidentified cell lines were used in this study. No commonly misidentified cell lines were used in this study. Specific accession numbers are provided for all primary cells used.
